# T3:T4 Ratio Can Distinguish Between Adaptive Changes and True Subclinical Hypothyroidism in Older Adults

**DOI:** 10.1093/geroni/igaa057.738

**Published:** 2020-12-16

**Authors:** Enoch Abbey, John McGready, Eleanor Simonsick, Jennifer Mammen

**Affiliations:** 1 Johns Hopkins University, Baltimore, Maryland, United States; 2 Johns Hopkins School of Public Health, Baltimore, Maryland, United States; 3 National Institute on Aging, Bethesda, Maryland, United States; 4 Johns Hopkins School of Medicine, Baltimore, Maryland, United States

## Abstract

Because of heterogeneity in hormonal aging,1 we believe isolated elevated TSH is insufficient to drive clinical decision making for thyroid hormone replacement in older adults. We performed a cross-sectional study involving 63 older adult participants of the BLSA in order to assess the diagnostic value of individual hormone levels or free T3: free T4 ratio for differentiating thyroid-aging phenotypes. We defined two phenotypic groups, central adaptation and primary hypothyroidism, both with a rising TSH and with a rising or falling FT4 respectively. Fifty-four percent of study participants were male, the average age was 78.8 years, and 66.7% had the primary hypothyroidism phenotype. The unadjusted odds ratio of having the central adaptation phenotype is 23.40 (95% CI 3.66-149.73) for every unit increase in the FT3:FT4 ratio. The ROC curve had a C-statistic of 0.815. Similarly, FT4 alone distinguished the phenotypes with a C-statistic of 0.864. In contrast, TSH and FT3 were not predictive (C-statistic of 0.617, and 0.479 respectively). When the analysis is limited to the 24 individuals with elevated TSH, the ratio remains predictive (0.839). Both the higher FT4 and the lower ratio found in individuals with adaptive changes are consistent with a physiology similar to the adaptations seen in acute illness. This supports the hypothesis that elevated TSH can represent a response to stressors with aging and doesn’t always warrant treatment with thyroid hormone. Our findings suggest that full thyroid function panel can be used to make better diagnostic decisions in older adults.

